# 1017. Gut Microbiota Diversity and Beneficial Metabolite Production is Reduced in Liver Transplant Recipients and Associated with Post Operative Infection

**DOI:** 10.1093/ofid/ofab466.1211

**Published:** 2021-12-04

**Authors:** Christopher J Lehmann, Robert Keskey, Matthew Odenwald, Ravi Nayak, Maryam Khalid, Eric Littmann, Eric G Pamer, Eric G Pamer, Talia Baker

**Affiliations:** 1 University of Chicago Medicine, Chicago, Illinois; 2 University of Chicago, Chicago, Illinois

## Abstract

**Background:**

Liver transplant (LT) recipients have abnormal microbiota before and after transplantation. (1,2) Associations between fecal microbiota, microbial metabolites, and clinical outcomes in liver transplantation are not well established. We correlated fecal microbiota composition and metabolite concentrations with early LT outcomes, including infection.

**Methods:**

In a prospective observational study, we collected peri-transplant fecal samples and determined microbiota composition by 16S ribosomal RNA gene sequencing in LT recipients. Fecal short chain fatty acid (SCFA) and bile acid concentrations were measured by targeted GC- and LC-MS analyses, respectively. Inverse Simpson index was used to determine microbiota alpha-diversity in subjects and healthy controls. Clinical outcomes including length of stay, ICU admission, liver function, antibiotic use, immunosuppressive requirement and post-operative infection were correlated with microbiota composition.

**Results:**

69 patients were enrolled, 70 liver transplants were performed and 307 peri-transplant fecal samples were collected and analyzed. Compared to healthy controls, the fecal microbiota of LT recipients had reduced alpha-diversity (p< 0.001). [Fig1] Bacteroidetes, Ruminococcaceae, and Lachnospiraceae, three taxa associated with a health-promoting microbiota, and their metabolites, SCFA and secondary bile acids, were markedly diminished 55% of LT patients.(3) Intestinal domination ( >30% frequency) by Enterococcus or Proteobacteria species was common and occurred in 36% of LT recipients. 76 post-operative infections occurred in 40 LT recipients, with Enterococci causing 52% and Proteobacteria 41% of bacterial infections. In subjects with fecal samples collected within 5 days of infection, 9/17 infections were caused by the organism dominating the microbiota. [Fig2]

Microbiota Composition and Metabolite Production

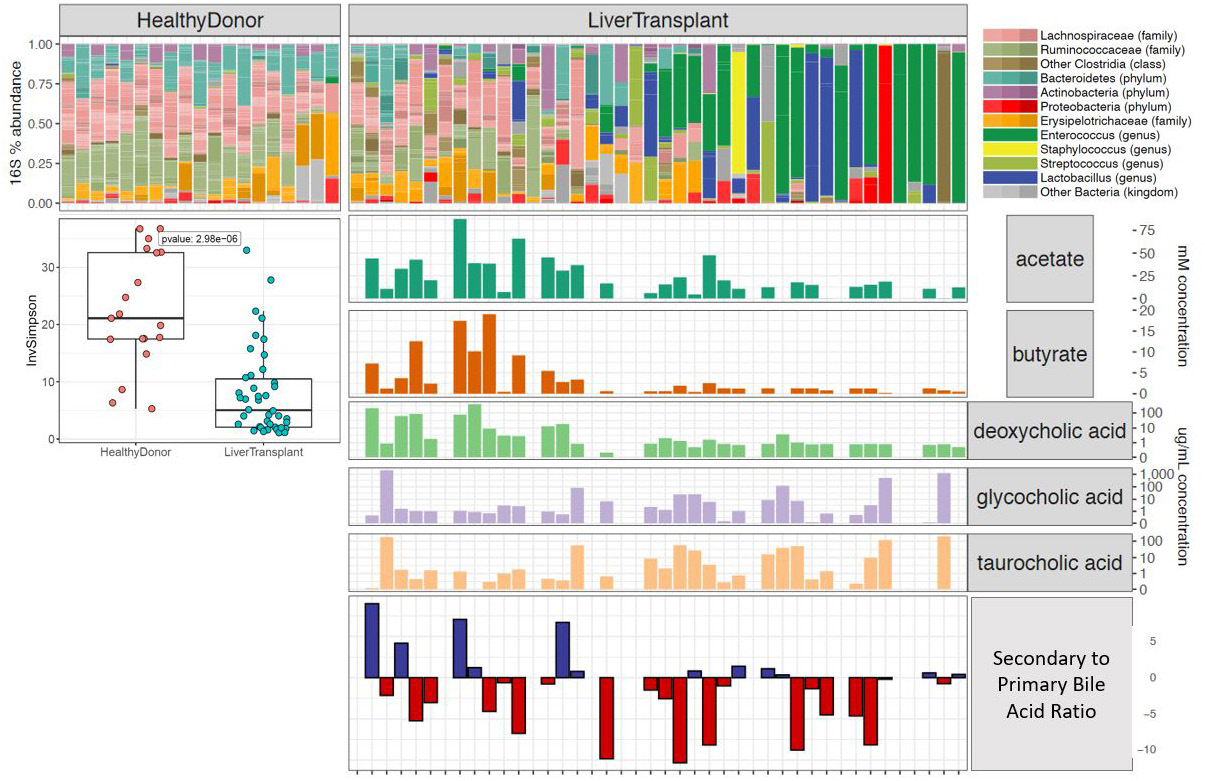

16s gene sequencing color coded by taxonomy. Each bar represents one stool sample nearest to LT compared to healthy controls. Alpha diversity measured by inverse simpson index. Absolute values of microbial metabolites and ratio of primary to secondary bile acids.

Comparison of Microbiota Composition and Post Operative Infection

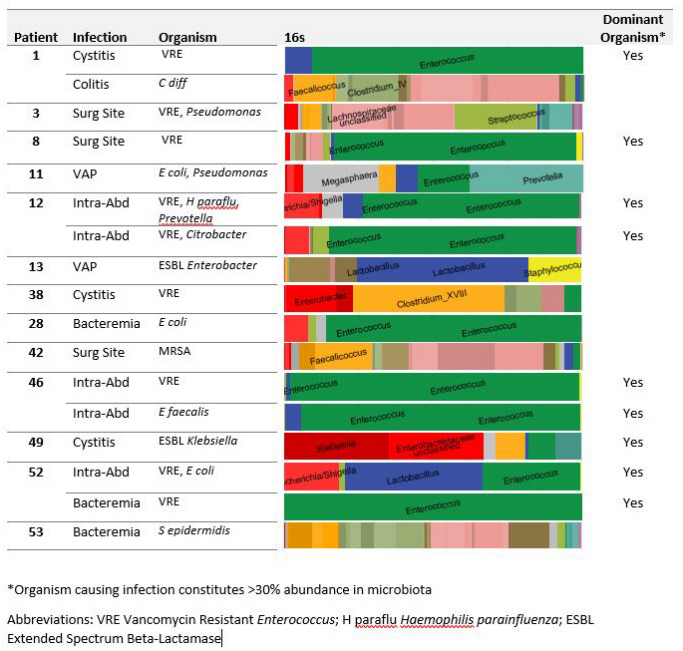

All bacterial infections captured with a microbiota sample within 5 days of infection.

**Conclusion:**

Microbiota diversity and microbially derived metabolites are markedly reduced in >50% of LT recipients. Intestinal domination and post-operative infections caused by antibiotic-resistant Enterococcus and Proteobacteria correlate with loss of Bacteroidetes, Ruminococcaceae, and Lachnospiraceae species, suggesting a potential role for microbiota reconstitution therapy in LT patients.

**Disclosures:**

**Eric G. Pamer, MD;FIDSA**, Nothing to disclose

